# The Use of Gutta Percha as a Root Canal Filling

**Published:** 1892-09

**Authors:** Forest G. Eddy

**Affiliations:** Providence, R. I.


### The Use of Gutta-Percha as a Root-Canal Filling.

BY FOREST G. EDDY, D.M.D., PROVIDENCE, R. I.

Read before the Vermont State Dental Society, March, 1892. Published in the *International Dental Journal*, March, 1892.

I bring before the society for discussion to-night my experience with gutta-percha as a filling for root-canals. I claim no originality, but rather present my gleanings from the operations of those in our profession who have made a success in the filling of root-canals and the saving of the natural teeth in a healthy condition. Some one has said: "Success in preserving devitalized teeth depends almost solely upon the individual skill of the operator, and possibly somewhat upon the materials used." To me the material used is a marked factor in relation to success.

We, who live in malarious districts, are beset in our practice with roots of teeth difficult of access, with their flattened and tortuous canals filled with pulps exposed, disintegrating and suppurating. To overcome these troublesome members, and bring them under subjection in as short a time as possible, is no slight strain upon the already overtaxed nerves of the busy dentist.

Here you have the most common methods: Root-canals with a drilled vent—a discharging sewer. Root-canals with pulp removed and not filled—a catch-basin for sewage. Again, canals filled with cotton, with iodoform, wood, lead, tin, oxychloride of zinc, gold and gutta-percha. Skilled operators have made a success in the use of all these methods and materials in filling the canals of teeth.

The material that is simple in its manipulation, the method that may be acquired by the majority, should rank first.

Gutta-percha seems paramount in value. The nature of this substance and its properties have been well described to us by our associate, Dr. Meriam—its non-elasticity; its wood-like hardness and toughness when cold; its being soft and easily moulded at high temperatures; its insolubility in water, alcohol, dilute acids and alkalies; its ready solubility in bisulphide of carbon, essential oils, and chloroform. I think it advantageous to use it in both conditions, hard and in solution,in the filling and closing of canals: hard, in the form of small pellets and cones; soft, dissolved in chloroform, which is the method in common use. Then to this solution of gutta-percha—"chloro-percha," as we call it—I add an equal bulk of oil of eucalyptus and oil of cassia,—the essential oils holding the gutta-percha in solution after the chloroform has to some extent evaporated. You all know how difficult it is to carry chloro-percha into canals from the rapid evaporation of the chloroform, leaving the gutta-percha sticking to the instrument rather than to the walls of the cavity.

A prominent, an essential factor to success is that the root be in an aseptic condition. Operators differ in obtaining this result, as they do in the use of different materials in closing the apical foramen and filling the canals.

Dr. H. Storer How says: "All methods are defective in which the operator does not know that he has closed the apical foramen."

The antiseptics of to-day are familiar, and the list is constantly increasing. The foremost writers upon antiseptics now advocate abolishing, as far as possible, all escharotics and coagulants in the treatment of septic conditions of root-canals. No antiseptic in use by the dentist answers these questions so well as peroxide of hydrogen and the essential oils. They are non-escharotic, non-toxic,—yet antiseptic and stimulant; the manner of their use is simple and positive.

In case of immediate extirpation of the pulp by means of forcibly inserting a plug of wood,—this being preceded by an injection of cocaine, or the patient being under the influence of nitrous oxide gas,—I at once wash the cavity with hot water, to stop the hemorrhage; dry out the canal with cone of bibulous paper, in the manner described by Dr. Smith Dodge, of New York; churn out the canal with peroxide of hydrogen, instead of carbolic acid, as an antiseptic; then re-dry the canal and fill with solution of gutta-percha in essential oils, which is easily worked to the apex and adheres to the walls.

Into the canal filled with the solution, carry a small piece of warm gutta-percha, gently and firmly, to the apex, following with the hard cone. The surplus solution will be forced out, and the hard cone of gutta-percha will be cemented to the walls of the canal. It will be seen that I differ here from Dr. How, if I may be permitted to again quote from his article, in which he says: "The fluid or soft plastic methods are defective, because it is only supposed, but not known, that the foramen is, in fact, tightly closed, to say nothing of the mischief likely to follow the probable forcing of the solution through the foramen."

If the pulp be in a suppurative and disintegrating condition, we remove the debris, using the peroxide of hydrogen from time to time to clean the cavity as we proceed, and dress the root with fibre of cotton or silk, saturated with oil of cassia or other essential oils.

When the root is in a healthy condition, fill with gutta-percha in the manner before described.

The root having a fistulous opening, the canal is cleared of debris, and filled with peroxide of hydrogen. By a piece of soft unvulcanized rubber and a blunt instrument used as a piston, the peroxide is forced through the canal and out of the fistulous opening, the whole tract being left in an aseptic condition. After again drying the canal, the solution of gutta-percha is forced in similar manner through the fistulous opening, closing the canal, as before, with gutta-percha.

Frequently, after filling the canal by some of the old methods, a slight discharge would continue from the fistulous tract; but never have I had a case that would not yield to the above treatment. My theory is that the old sac at the apex of the root is distended and filled with the solution of gutta-percha, and this remains encysted. Some inflammation often results from forcing the solution of gutta-percha through the fistulous tract, but in a day or two at most it usually subsides.

In the treatment of root-canals I have used the peroxide of hydrogen for eight years faithfully, it being first brought to my attention by Dr. Chittenden, of Madison, Wis. I have used gutta-percha in different and various forms for about the same length of time.

From working upon and refilling canals that have had cotton, oxychloride of zinc, wood and metallic stoppings, I am led to think of gutta-percha as occupying the foremost position in the closing of roots of teeth that are devitalized.

I have taken the liberty, and I know you will allow it to me, to bring a few extracts from the records of work done in my office upon devitalized teeth.

From November 1, 1890, to November 1, 1891, a period of twelve months, I find the canals of one hundred and fifty-two teeth successfully filled after the manner described. To classify them a little more thoroughly, there were fifty-seven molars, fifty-seven bicuspids, eight cuspids, seventeen lateral, and thirteen central incisors. During the same length of time but two were removed as complete failures. Both of these were most faithfully operated upon, and I consider their loss and failure due to the low vitality of the patients.

I am led to advocate this method because the antiseptics as used are not coagulants, and are not escharotic; because of their non-irritating character in relation to soft tissues; and because of their pleasant odor in the office. No creolin, creosote, or iodoform is used in my office.

